# Evidence for Emergency Vaccination Having Played a Crucial Role to Control the 1965/66 Foot-and-Mouth Disease Outbreak in Switzerland

**DOI:** 10.3389/fvets.2015.00072

**Published:** 2015-12-14

**Authors:** Dana Zingg, Stephan Häsler, Gertraud Schuepbach-Regula, Heinzpeter Schwermer, Salome Dürr

**Affiliations:** ^1^Veterinary Public Health Institute, Vetsuisse Faculty, University of Bern, Bern, Switzerland; ^2^Swiss Association for the History of Veterinary Medicine, Gasel, Switzerland; ^3^Federal Food Safety and Veterinary Office, Bern, Switzerland

**Keywords:** foot-and-mouth disease, control strategies, Switzerland, model validation, historic data, emergency vaccination, DADS

## Abstract

Foot-and-mouth disease (FMD) is a highly contagious disease that caused several large outbreaks in Europe in the last century. The last important outbreak in Switzerland took place in 1965/66 and affected more than 900 premises and more than 50,000 animals were slaughtered. Large-scale emergency vaccination of the cattle and pig population has been applied to control the epidemic. In recent years, many studies have used infectious disease models to assess the impact of different disease control measures, including models developed for diseases exotic for the specific region of interest. Often, the absence of real outbreak data makes a validation of such models impossible. This study aimed to evaluate whether a spatial, stochastic simulation model (the Davis Animal Disease Simulation model) can predict the course of a Swiss FMD epidemic based on the available historic input data on population structure, contact rates, epidemiology of the virus, and quality of the vaccine. In addition, the potential outcome of the 1965/66 FMD epidemic without application of vaccination was investigated. Comparing the model outcomes to reality, only the largest 10% of the simulated outbreaks approximated the number of animals being culled. However, the simulation model highly overestimated the number of culled premises. While the outbreak duration could not be well reproduced by the model compared to the 1965/66 epidemic, it was able to accurately estimate the size of the area infected. Without application of vaccination, the model predicted a much higher mean number of culled animals than with vaccination, demonstrating that vaccination was likely crucial in disease control for the Swiss FMD outbreak in 1965/66. The study demonstrated the feasibility to analyze historical outbreak data with modern analytical tools. However, it also confirmed that predicted epidemics from a most carefully parameterized model cannot integrate all eventualities of a real epidemic. Therefore, decision makers need to be aware that infectious disease models are useful tools to support the decision-making process but their results are not equal valuable as real observations and should always be interpreted with caution.

## Introduction

Foot-and-mouth disease (FMD) is one of the most contagious animal diseases of cloven-hoofed animals with high economic impact (1). The agent, the FMD virus, can be distinguished in seven serotypes and these further in numerous subtypes (1). The serotypes A, O, and C caused three big epidemics in Switzerland in the last century (2).

**Figure 1:**
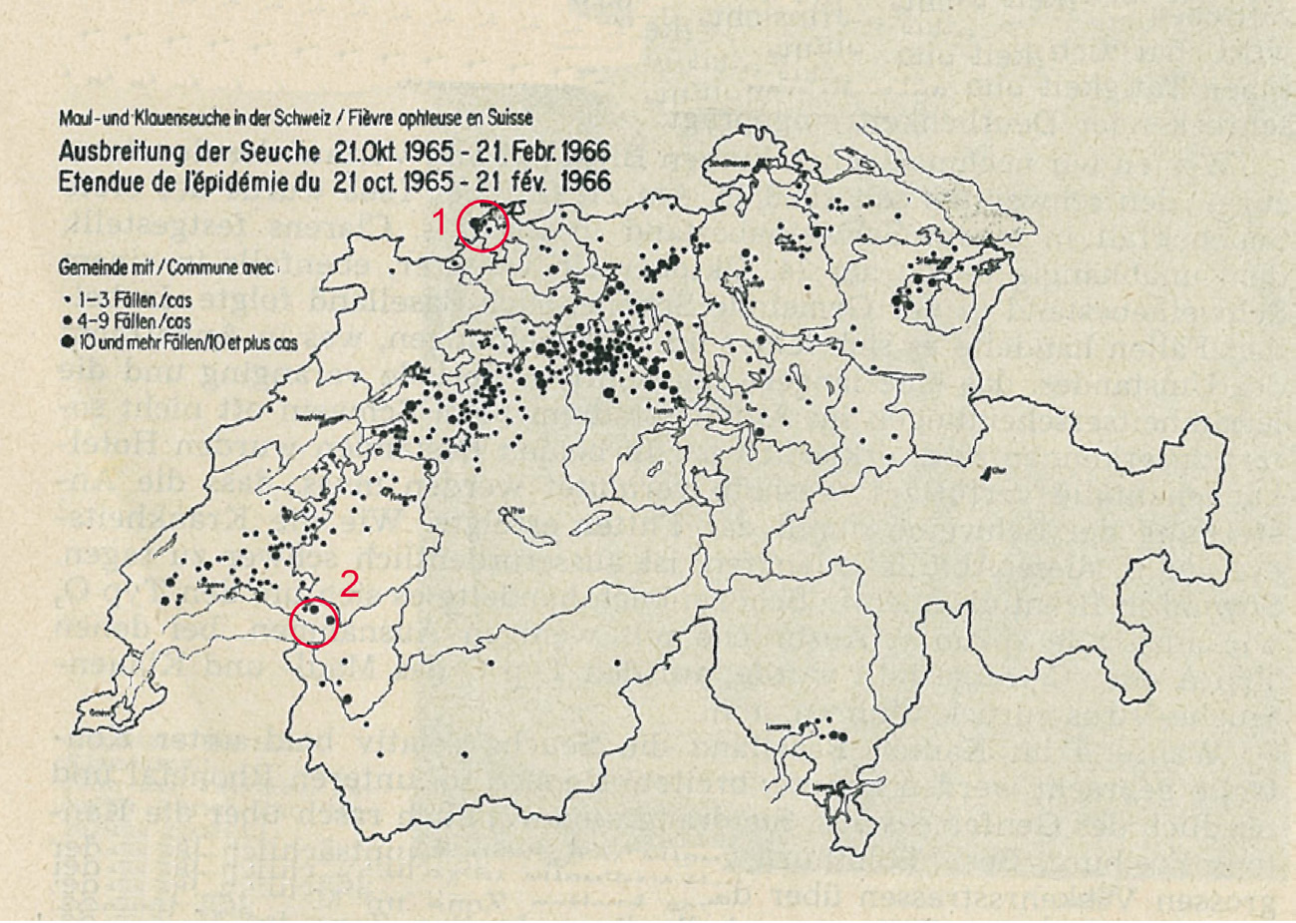
**The spread of the foot-and-mouth disease epidemic from the 21 October 1965 to the 21 February 1966 in Switzerland (map includes lakes and cantonal borders)**. Source: archives of the Federal Food Safety and Veterinary Office. The legend lists the affected communities in three categories pictured by different sizes of the points: 1–3 cases (smallest), 4–9 cases, and 10 or more cases (largest). The red circles symbolize the two index case villages (1, Schönenbuch; 2, Brent).

The last of these epidemics emerged in 1965/66 and spread over large part of Switzerland (Figure 1). It was caused by the strain O_1_ (serotype O, subtype 1), which was later called O_1_-Lausanne (3). Only few FMD cases had been reported before the epidemic during early 1965. These were mostly found in eastern Switzerland. All of these cases were caused by the serotypes A or C (communication of the Federal Veterinary Office, volumes 34–52, 1965). On 21st of October 1965, the first case of FMD, caused by the serotype O_1_, was reported in Brent, canton of Vaud in western Switzerland. The second case came to light just 3 days later in Schönenbuch, canton of Basel-Landschaft (4). In both cases, pigs were affected first. These pigs were fed with kitchen garbage from nearby hotels. Therefore, it was assumed that the virus had been imported via contaminated meat as the disease caused several epidemics in European countries (for example, Spain, Hungary, or Austria) at that time (2). The outbreak in canton Basel-Landschaft was brought under control rapidly by slaughtering all livestock present on the infected premises (IP). In the meantime, despite the slaughtering of animals on IPs, the disease spread from the outbreak in Brent to the region around the Lake Geneva and further afield to affect nearly the whole Swiss lowland with a few cases recorded in the alpine mountain region (4). The peak of new cases was reported at the end of December 1965. After vaccinating two-thirds of the entire cattle population by the end of 1965, and the remaining third by the middle of January 1966, the number of new cases slowly decreased (communications of the Federal Veterinary Office, volumes 1–14, 1966). However, because pigs had not been vaccinated a new “pig-FMD epidemic” developed in canton of Lucerne (4). This autonomous epidemic could only be stopped after implementation of a vaccine, developed especially for pigs, which was used in addition to the monovalent vaccine applied before. The epidemic was brought under control by the end of March 1966 (4). However, sporadic cases have been observed thereafter until June 1966 from when onward no FMD case was recorded until early December 1966 (4).

The control measures taken in this FMD epidemic did not differ greatly from the measures foreseen in the current Swiss and European contingency plan, disregarding the slaughtering instead of the nowadays culling (Council directive 2003/85/EC of 29 September 2003 on Community measures for the control of FMD). They consisted of slaughtering of all animals on IPs in determined slaughterhouses, epidemiological surveillance, movement restrictions, cleaning, and disinfection on the IPs (4). The meat was sold for consumption after treatment with lactic acid. In addition to the current default measures, a ring vaccination was implemented. Control, surveillance, and vaccination zones were assigned according to local conditions, taking the landscape, localization of farms, or villages and infrastructure into account. At that time, no explicit size of the zones was required by the Swiss legislation. Even though ring vaccination was applied intensively, it could not prevent the disease from spreading further, which was the reason why the entire cattle population was vaccinated by January 1966. Once mass vaccination was completed, slaughtering was limited to non-vaccinated animals and animals showing clinical symptoms (4). Vaccinated animals on IPs without clinical signs were revaccinated if the time period since the last vaccination was longer than 12 days (Conference of Cantonal Chief Veterinarians, 1966).

Retrospective view on the data suggests that the country-wide cattle and pig vaccination was the crucial intervention to control the 1965/66 FMD epidemic and finally eradicated the virus. However, no comparable dataset is available to investigate this hypothesis. What would have happened without vaccination? Nowadays, computer-based simulation models are useful tools to investigate control measures and impacts of theoretical epidemics. In the present study, the spread of the 1965/66 FMD epidemic in Switzerland with and without vaccination was simulated and compared.

Stochastic computer models exist since the 1950s (5). Different FMD models have been developed by international research groups, including InterSpread (6, 7), Auspread (8, 9), North American Animal Disease Spread Model (NAADSM) (10), Exodis FMD (11), the Warwick model (12, 13), and Davis Animal Disease Simulation (DADS) model (14). Epidemiological models were used in numerous countries (e.g., Denmark, USA, Switzerland, UK) to simulate epidemics on FMD and to investigate benefit of vaccination or cost savings of different control measures (14–22). Furthermore, decision support tools have been developed based on simulation results (23). While some of these models were used based on real outbreak data [e.g., the FMD outbreak in 2001 in Great Britain (12, 24) or 2010 in Japan (25)], others were generated for FMD-free countries integrating detailed information on agricultural practices and population structure (8, 21).

In the present study, the DADS model was applied, which has been modified from an earlier study on FMD epidemics in Switzerland (21). The objectives of the present study were to evaluate how well the DADS model replicates the 1965/66 FMD epidemic and to evaluate the effectiveness of the vaccination strategy applied during that outbreak. The study combined current modeling techniques and historic outbreak information, in order to inform policy makers for decisions on how to control FMD outbreaks in the future.

## Materials and Methods

### Swiss Livestock Premises in 1966

Switzerland consists of 26 cantons which, in 1966, were further subdivided into 3085 communities. These communities correspond to one or few postal code areas depending on the size of the community.

Data on livestock population were available from a census conducted in 1966, which was obtained from the Swiss Federal Statistical Office. The data were manually entered into Microsoft Excel, because the censuses were only digitized after 1975. For each livestock species, the number of owners per community was recorded. The total number of premises per community was not available. Therefore, a farmer who owned pigs and cattle was counted twice, as a cattle owner and as a pig owner. The number of animals per species per community was also available from the livestock census. Additionally, the percentage of farms with different herd size categories per species was available on cantonal, but not community, level. Recorded categories for cattle consisted of 1–4, 5–10, 11–20, and >20 animals, for pigs of 1–3, 4–10, and >10 animals, and for small ruminants of 1–5, 6–25, and >25 animals.

Demography data had therefore to be reconstructed. In a first step, the number of premises per community and premises type was calculated. Premises types were defined based on the farmed species while any possible combination of the four species (cattle, pig, sheep, and goat) with one to four different species on each premises was considered (Table 1). As no data on farm structure in 1965/66 were available, contemporary witnesses were interviewed to estimate the relative distribution of mixed versus single-species farms. They answered consistently that in 1965/66, there were few premises that kept only one species of cloven-hoofed animals. The majority of premises farmed cattle and pigs and/or small ruminants. Cattle and pig were the most important productive livestock, while small ruminants played a minor role. First, based on the number of livestock owners per species and community and the assumption that cattle–pig premises were predominant, the number of the cattle–pig premises per community was equal to the number of cattle or pig owner, whichever was smaller. Second, if there were more cattle than pig owners in a community, the goats and sheep owners were allocated to the remaining cattle owners resulting in mixed cattle–sheep or cattle–goat premises, respectively. Vice versa, if more pig than cattle owners were situated in a community, the sheep and goat owners were allocated to remaining pig owners resulting in pig–sheep or pig–goat premises, respectively. Third, if goat and sheep owners were left, they were classified as small ruminant (sheep–goat) premises. Finally, all remaining owners were either counted as single species or cattle–pig-small ruminant premises based on the number and type of owners remaining. The output of this allocation was a list of the number of 15 different premise types per community (Table 1, summarized overall Switzerland).

**Table 1:** **Number and size of premises per type of the reconstructed livestock population in Switzerland based on the census of 1966**.

Premises type	Number of premises (total 138,587)	Number of animals	
		Range	Median
Cattle	15,283	1–76	12
Pig	2645	1–382	5
Sheep	4575	1–1801	6
Goat	4631	1–97	2
Cattle–pig	81,660	2–524	24
Cattle–sheep	11,319	2–432	19
Cattle–goat	8837	2–151	12
Pig–sheep	463	2–148	13
Pig–goat	212	2–62	7
Sheep–goat	2664	2–489	15
Cattle–pig–sheep	1456	3–153	26
Cattle–pig–goat	1757	3–130	10
Pig–sheep–goat	47	3–71	14
Cattle–sheep–goat	361	3–116	21
All four species	2677	4–176	26

In a second step, the number of animals per species and community was randomly distributed to the premises. From the livestock census, the percentage of different herd size categories per species was available for each canton. Principally, the animals were distributed to the premises proportionally to those reported herd size categories. If this cantonal distribution did not fit to a community (e.g., if a community had an extraordinary high number of small premises compared to the average in the same canton), the animals were distributed equally to all premises in the given community. This procedure resulted in herd sizes of 1–1801 animals (Table 1).

In a third step, since only the community, and not the exact geographic locations, of the premises was known in 1966, the coordinates (WGS 84) were generated randomly in the corresponding postal code area in which the premises were located. Lakes and areas higher than 1500 m were omitted, as the 1966 FMD outbreak occurred in winter when alpine premises were unpopulated. These coordinates were assigned randomly to the reconstructed premises (Figures S1 and S2 in Supplementary Material). The coordinates for 26 communities had to be generated manually because they were located extraordinary high (>1500 m), and nevertheless inhabited throughout the year.

### Collection of Historical Data

The incidence reports (weekly number of new outbreaks per community) of the 1965/66 FMD outbreaks were obtained from the Swiss Federal Veterinary Office. The original reports, with details of the IPs including the number of infected cattle or pigs, are archived in the Swiss Federal Archives. Unfortunately, these records were not complete, especially in the time period from December 1965 to January 1966, when the majority of cases occurred and personnel resources were limited to record them completely. Nevertheless, these data informed parameter estimations such as shipment size, farmer compliance to movement restrictions, or limitations for the number of slaughtered animals per day due to limited personal and material resources. Additionally, reports from Conference of Cantonal Chief Veterinarians, the annual reports of the Swiss Federal Veterinary Office, FMD reports to the Swiss Minister of Economy and the States Archive of the canton of Zürich were used to gain insight into agricultural practices at that time to better estimate model parameter values. Furthermore, additional background information about agricultural life and farming practices in the 1960s has been collected from several experts, who were contemporary witnesses, virologists, and former veterinarians and former employees of the Federal Vaccine Institute, by personal interviews.

### The Simulation Model

The DADS model was developed to simulate FMD outbreaks in California, using R environment software (https://cran.r-project.org/) (14, 26). It is a stochastic, spatiotemporal model, which allows simulations of the spread of a disease among spatially explicit premises. At the start of the simulation, the disease is introduced to one or more premises. The infected animals pass through a latent, subclinical infectious, clinical infectious, and immune stage if not culled earlier. The time they spend in one of these stages is sampled from user-defined distributions. All animals on IPs are eventually culled. The time step used by the model is one day. Direct contact (DC, animal movement), high-risk indirect contacts (HRIC, e.g., via veterinarians or cattle dealer), and low-risk indirect contacts (LRIC, e.g., via visitors, virus spread via visits of cheese dairy or restaurants) are considered by the model. Also, local area spread (including airborne spread), containing short distance disease spread not covered by DC or IC is included in the model. Control measures such as culling, movement restrictions, and vaccination can be applied by the user. All input parameters of the model with their values are used in this study and the sources of information are presented in supplementary Table S1 in Supplementary Material.

Here, the DADS model already adapted earlier to the Switzerland (21) was modified in five ways. First, the time delay between the detection of IP and culling was introduced as a step function instead of a fixed value. If the number of animals to cull exceeded a certain threshold, the time delay from diagnosis to culling increased. This reflects the fact that personal and transport capacities are limited and critical during time periods with a high incidence. Consequently, time to cull and infectious period for individual animals increased, which may enlarge the epidemic. Second, three parameters (“time to extended vaccination cattle,” “time to extended vaccination pig,” and “larger vaccination radius”) were integrated to permit a nation-wide vaccination from a user-defined point in time during the outbreak. The former two parameters reflect the point in time when nation-wide vaccination started in 1965/66, which differed between cattle and pig premises. The latter parameter is an extended vaccination radius that results in a nation-wide vaccination. The parameter defining the premises types to be vaccinated also had to be adapted when the pigs were added as targeted species for vaccination. Third, the parameters “time vac all cattle,” “time vac all pig,” and “larger vaccination radius2” were defined. The first and second parameters were used to define a point in time when state-wide cattle and pig vaccination, respectively, should be completed. At this point in time, all premises with cattle (or pig) that have not been vaccinated yet were vaccinated in a radius that was defined by the last parameter. Fourth, the shipment size (number of animals per DC to other premises) was defined for each premises type individually as usually the number of animals shipped depends on the species (size of vehicle, herd structure). Cattle and goat were often shipped as individuals, whereas pigs and sheep were usually shipped in groups. Finally, the epidemiological phases were defined for each premises type individually, as the latent, subclinical, and clinical periods of FMD depend on the species.

Sales yards were not included in the model. It was assumed that markets and local sales yards in the affected regions were closed due to the outbreak, and therefore their impact on the epidemic was assumed to be negligible.

### Model Parameterization for Switzerland in 1965/66

The aim of this study was to evaluate how well the model will perform for a prediction and evaluation of control strategies of future outbreaks. Because outbreak data will not be available in future epidemics, parameter values will have to be estimated based on best knowledge from varying sources (notably data on livestock premises and transports, literature, and expert opinion). Therefore, also in this study, most parameter values were defined based on diverse sources of information (expert opinion, historical reports, literature) rather than fitted to the outbreak data (Table S1 in Supplementary Material). Input parameters are categorized as general input, intra-premises spread, inter-premises spread, and control strategy parameters. Values for general parameters were gathered from historical sources and expert opinion. The model simulation time was set to 2 years to allow substantially longer outbreaks than in 1965/66 (which lasted 150 days). As in 1965 FMD was endemic, it was assumed that the disease awareness was much higher and farmers would have recognized clinical signs in about a week – earlier than today where it was found to be on average 14–15 days, although with a high variability between observed epidemics (27). FMD already was a notifiable disease in Switzerland during the time of the outbreak and, therefore, it can be assumed that most cases were reported readily after detection. Based on expert opinion and information derived from the protocol of a conference of the World Animal Health Organization in Rome after the epidemic (in March 1966), the initial diagnosis delay was set at 7 days. Once the first case has been detected, the diagnosis delay was assumed to be reduced to 2–5 days, because disease awareness increased. Intra-herd transmission parameters are generic and were sourced from the literature (14). Inter-herd transmission parameters, i.e., probabilities of FMD transmission given a contact, were adopted from earlier work on FMD simulation in Switzerland (21, 28). Daily rates of DC, LRIC, and HRIC were based on the statements of six contemporary witnesses who were interrogated on the frequency of contact types between premises (animal shipment, veterinarian visits, visits from other farmers, indirect contacts via cheese dairy, restaurants, or church visits). Parameter values differed between contact type and premises type (Table S1 in Supplementary Material). Contact rates of mixed premises (more than one species) were assumed the same as those for pig or cattle premises, respectively, if one of these species was present on the premise. For all other mixed premises, the contact rates for the species with the highest contact rates were assigned. Distributions of distances for DC and IC were defined based on personal interviews of the six contemporary witnesses and, for DC, compared with historic outbreak data. Statements of the witnesses revealed that DC predominantly occurred within villages and communities (up to 25 km) but also beyond with decreasing probability for larger distances. This information was incorporated into the model by visually fitting the DC distances to a Weibull distribution. The distance distribution of the HRIC was defined as 50% of the contacts occurred within 4 km (within a village), one-fourth within a community (up to 25 km) and one-fourth beyond (up to 50 km). The most relevant LRIC was stated to occur via cheese dairies. Their catchment area was estimated to be up to 10 km in the 1960s in Switzerland. The distance distribution of LRIC was, therefore, defined as uniform between 0 and 10 km. For 9.8% (4/41) of the parameters, information of any source, including expert opinion, was lacking, and values had to be based on assumptions (Table S1 in Supplementary Material). These were (a) the number of infected animals in the index herd (a pig premises) that was set at 100 out of 120 pigs as contaminated feed was expected as source of infection that can simultaneously infect all fed animals, (b) the time period needed to vaccinate the premises within a given radius that was assumed to be 8 days based on discussion with veterinary authority, (c) the time period between the detection of the first clinical case and the delivery of the vaccine that was assumed to be 14 days based on discussion with veterinary authority, and (d) the assumption that DC between premises were only possible with equal probability when the same species were present in both the sender and receiver premises.

Parameter values for control measures were mainly informed by experts. The duration of control zones was implemented as described in the minutes of the Conference of Cantonal Chief Veterinarians of January 1966, even though experts reported that it was not always respected. The diameter of control zones was estimated based on outbreak reports from the States Archives of the canton of Zurich and by information from contemporary witnesses. Vaccination effectiveness was sourced from the minutes of the Conference of Cantonal Chief Veterinarians of January 1966 and expert opinion.

### Model Simulations and Data Analysis

Basic control measures consisted of culling of all IP within a specific delay after FMD detection (stamping out), implementation of surveillance (6 km around IP), and protection (4 km around IP) zones with restricted animal movements for 21 days for both zones after stamping out of the triggering IP and tracing backward and forward. Premises targeted for culling are depopulated within a given time frame after detection, defined by the stochastic culling delay parameter. The culling delay was set at 2–3 days, and increased to 5–6 days for days when more than 200 premises are allocated for culling in a day.

The vaccination was implemented in four steps. The first step was vaccination of premises that kept cattle within a radius of 5 km around IP starting 2 weeks after the detection of the first FMD case. The vaccination area was extended to a radius of 25 km starting to simulate nation-wide vaccination from day 55 onward. This was approximately the point in time when nation-wide vaccination was implemented during the 1965/66 outbreak [middle of December 1965 (4)]. The second step was to complete the nation-wide vaccination of all cattle premises at day 84 by changing the vaccination radius to 400 km (covering whole Switzerland) that resulted in the vaccination of the entire cattle population by the middle of January 1966. Third, as the pig population was also vaccinated in 1966, all premises types with pigs within a radius of 25 km around an IP were additionally vaccinated starting at day 85. In a fourth step, as previously done for cattle, all pig premises not yet vaccinated at day 100 were vaccinated at this day by increasing the vaccination radius 400 km. The efficacy of the vaccine on herd level was set at 80–90%.

To assess the impact of the vaccination as a control strategy, a scenario without vaccination (NV) was simulated, considering same measures otherwise. Model simulations were performed with 150 simulations each for vaccination (V) scenario and NV scenario. The number of simulations was based on a previous study where DADS was used to simulate FMD outbreaks in Switzerland (21). The simulations run over 730 days (2 years).

The duration of the outbreak and number of animals and premises culled were assessed for the V scenario and compared with the real outbreak data from 1965/66. The end of the outbreak was defined as the point in time when the number of culled animals fall below 14, the average number of animals reported of being slaughtered per day in 1966 due to FMD after the outbreak was controlled (communication of the Federal Veterinary Office, volumes 15–52, 1966). To analyze the agreement of the spatial spread of FMD between model and reality, the area and position of the minimum convex polygon (MCP, R package adehabitatHR) of the 10% most fitting simulated outbreaks in regard to the number of culled animals was compared with the MCP of the real outbreak in 1965/66. Additionally, duration of the outbreak and number of animals and premises culled for simulations with and without vaccination were compared using independent Wilcoxon signed-rank tests. All analyses were performed using R.

### Sensitivity Analysis

Thirteen parameters that were suspected to have the biggest influence on the model outcome were analyzed: number of index animals per herd, detection delay for index and secondary cases, frequencies of daily DC and IC and their distances, shipment size for DC, time period between detection of IP and culling (culling delay), time period between detection of IP and vaccination (vaccination delay), and vaccine efficacy. To perform the sensitivity analysis, the values were varied ±15% around the default value based on previous work (21). For stochastic parameters, the range of the default distribution was set at −15% and +15%, respectively. Each parameter was tested separately by performing 150 simulations per value while keeping the values of all other parameters at the default.

Three outcome variables were considered for sensitivity analysis: duration of the outbreak, number of animals culled, and number of premises culled. Independent Wilcoxon signed-rank tests were performed to compare the outcomes using different parameter values.

## Results

### Accuracy of Model Simulations and Effect of Vaccination

**Figure 2:**
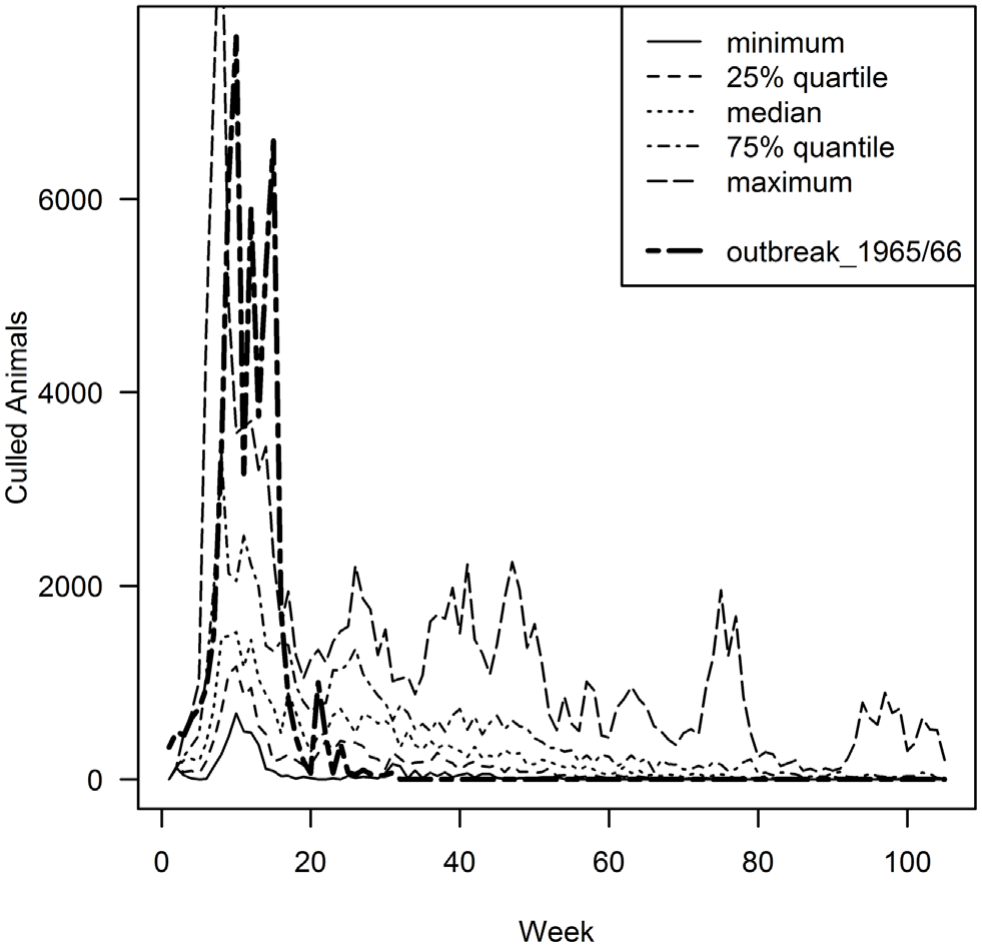
**Epidemic curve of the 1965/66 outbreak (bold dashed line) in comparison to the 10% (*n* = 15) largest simulated model outbreaks in relation to the number of animal culled, presented in quartiles**. Given that the outbreak data of 1965/66 only exists by week, the model output was scaled to weekly intervals.

**Figure 3:**
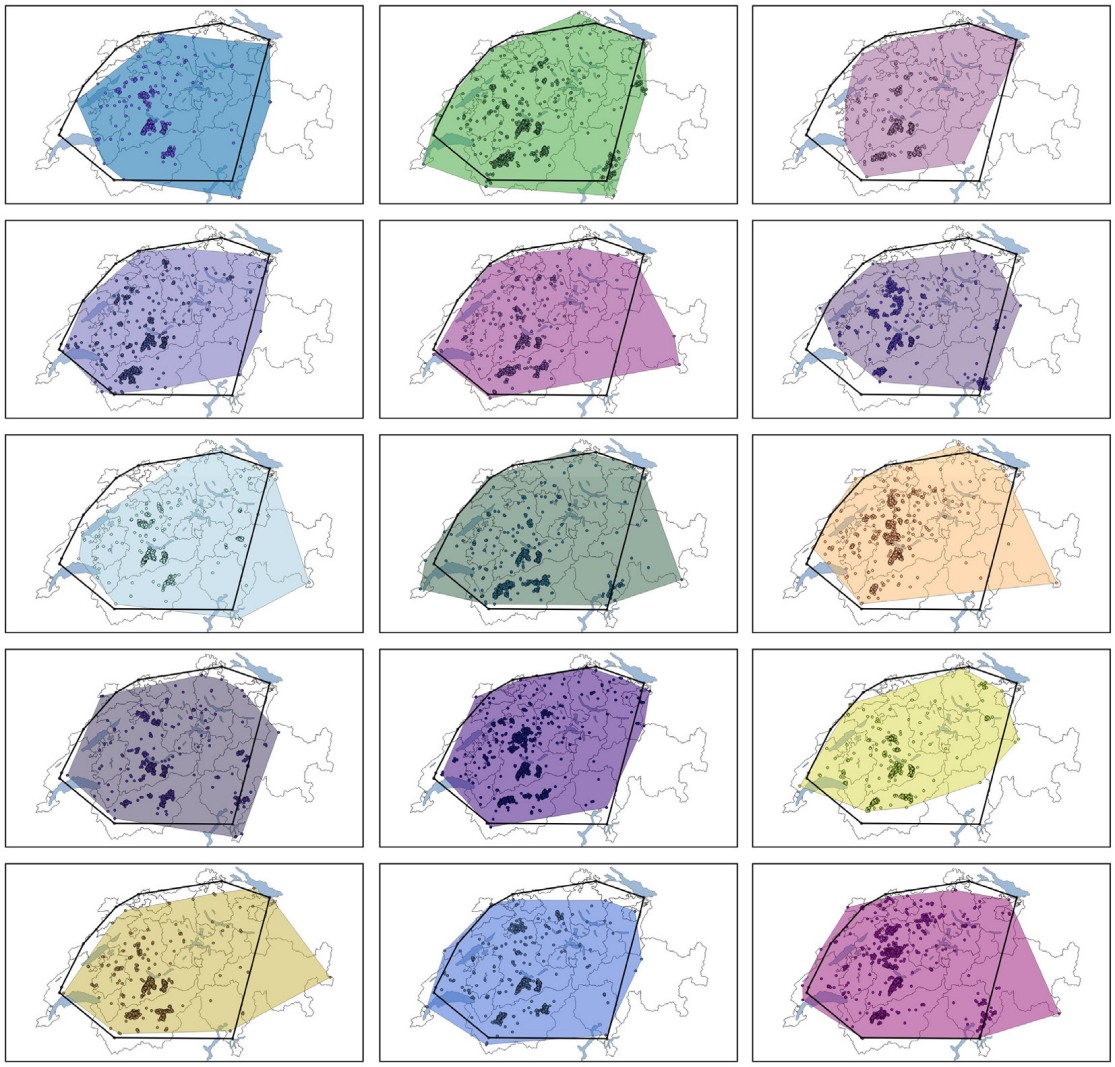
**Minimum convex polygon (MCP) of the 10% largest simulated outbreaks in relation to the number of animal culled (colored area) in comparison with the MCP of a FMD outbreak occurred in Switzerland in 1965/66 (black polygon)**.

By comparing the results of the model to the data of the real epidemic, only the largest simulated outbreaks (maximum 55,990 animals culled) approximated the observed number of slaughtered animals (51,215 animals were slaughtered in 1965/66) (Figure 2). Regarding the number of premises depopulated (942 in 1965/66), the largest simulated outbreak exceeds these numbers by 2238 premises (Table 2). The number of premises in which slaughtering took place in 1965/66 equates best to the 81.5 percentile of the model output (943 premises culled). The duration of the outbreak in 1965/66 could not be reproduced by the simulation model (Table 2; Figure 2). Taking into account the 10% largest outbreak simulations only, they all exceeded the duration of the observed outbreak by the factor of 3.5 at least. Particularly, after the peak of the epidemic in 1965/66 (week 21), the simulated epidemics continued, although at a low level (Figure 2). The 10% largest simulated outbreaks all covered an area similar to the area affected in 1965/66, which demonstrates that the nation-wide spread of FMD could be reconstructed by the model (Figure 3). The sizes of MCP area of the 10% largest simulated outbreaks, which ranged from 25,916 km^2^ to 40,177 km^2^ (medium 33,610 km^2^), were found to be similar to the one of the real epidemic (33,269 km^2^).

**Table 2:** **Summaries of predicted outbreak duration in days, number of culled animals and number of IP of 150 model simulations for the scenario with vaccination (V) and without vaccination (NV) in comparison with the recorded data of the 1965/66 FMD outbreak in Switzerland**.

	Outbreak duration		Animals culled		Infected Premises	
	V	NV	V	NV	V	NV
Minimum	12	12	123	129	1	1
25th percentile	25	24	249	285	5	6
Median	56	79	605	774	20	20
75th percentile	224	725	4088	865,785	176	35,818
90th percentile	520	553	30,381	974,246	1690	39,623
92.5th percentile	656	718	31,299	994,246	1876	40,272
95th percentile	714	724	32,400	1,012,856	2080	41,280
97.5th percentile	718	725	35,522	1,030,458	2378	42,240
Maximum	730	730	55,990	1,195,757	3180	47,122

*The outbreak duration of the 1965/66 epidemic was 150 days with 51,215 animals in 942 premises culled*.

**Figure 4:**
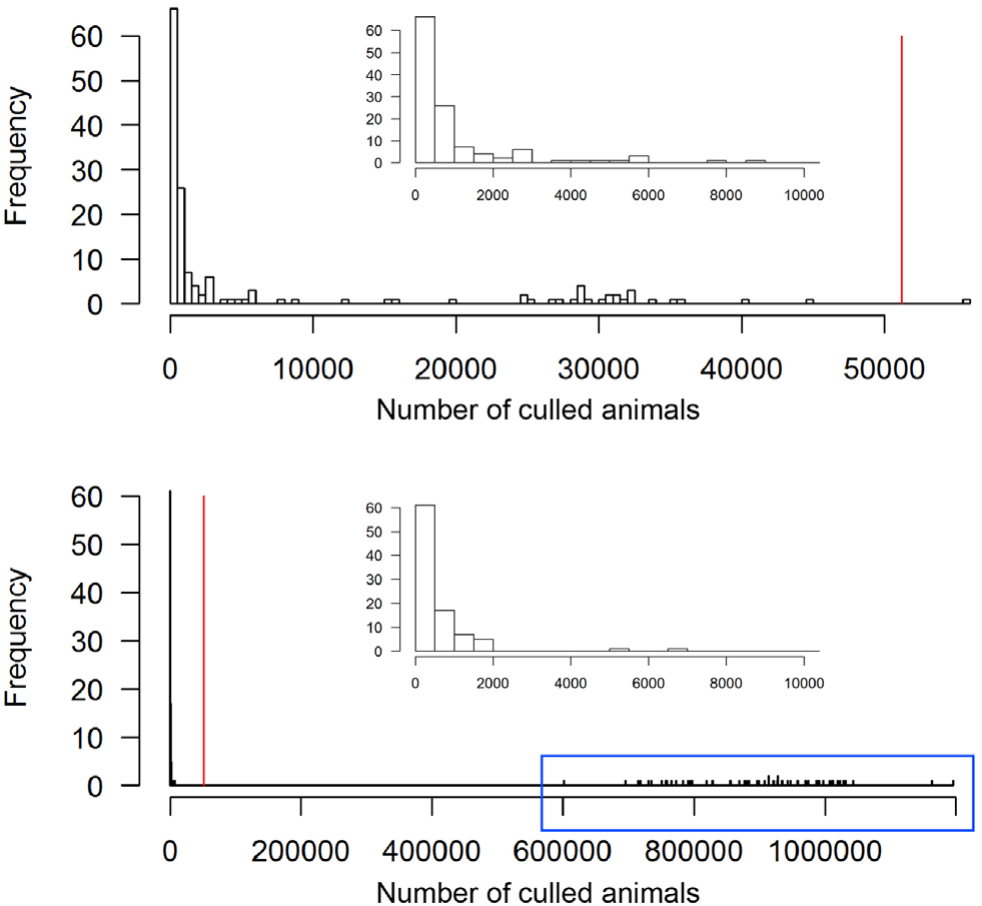
**Total number of culled animals of the 150 simulated FMD outbreaks in the Swiss livestock population in 1965/66 when vaccination was applied (top) and not applied (bottom)**. The red vertical lines represent the number of culled animals during the real FMD epidemic in Switzerland in 1965/66. The insets zoom into the range of outbreaks with less 10,000 animals culled. The outbreaks in the blue box of the non-vaccination strategy are those referred to as large outbreaks (*n* = 58).

**Figure 5:**
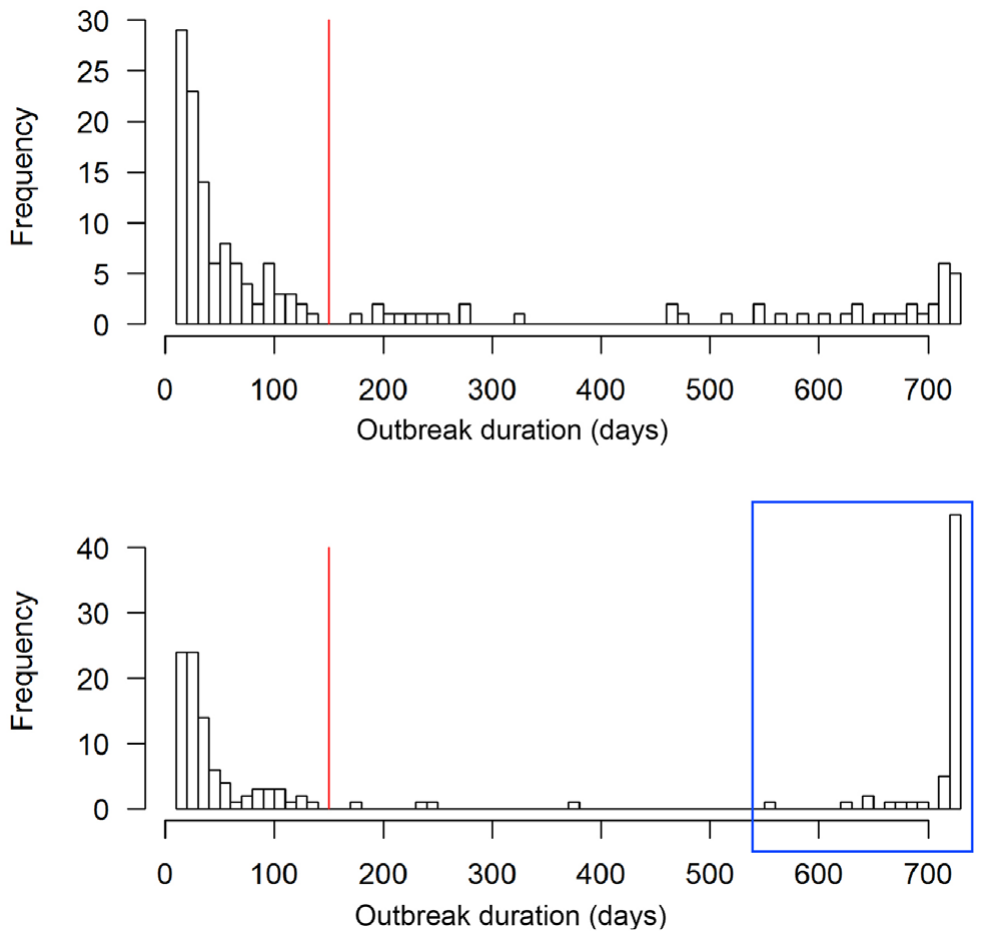
**Outbreak duration of the 150 simulated FMD outbreaks in the Swiss livestock population in 1965/66 when vaccination was applied (top) and not applied (bottom)**. The red vertical lines represent the outbreak duration of the real FMD epidemic in Switzerland in 1965/66. The outbreaks in the blue box of the non-vaccination strategy are those referred to as large outbreaks (*n* = 58).

The scenario without implementation of vaccination resulted in significantly higher numbers of animals and premises to cull compared to the vaccination strategy (*p* < 0. 0.002, Table 2). The outbreak duration was also significantly longer for the NV scenario (*p* = 0.01). The majority (61%, 92 out of 150) of the non-vaccination strategy simulations caused a similar number of animals culled as the vaccination strategy (Figure 4, insets). These outbreaks also had a similar outbreak duration distribution as when vaccination was applied (Figure 5). However, 58 out of the 150 simulations (39%) of the non-vaccination strategy became very large with a maximum number of animals culled of almost 1.2 Mio (Figure 4, bottom). They also were long lasting with 12 outbreaks not yet finished after 730 days (the maximum duration allowed in the model simulations) (Figure 5, bottom). The maximum of animals being culled for simulations where vaccination applied (almost 56,000 animals) was substantially smaller (Figure 4, top) and only four outbreaks were not yet finished after 730 days (Figure 5, top).

### Sensitivity Analysis

**Figure 6:**
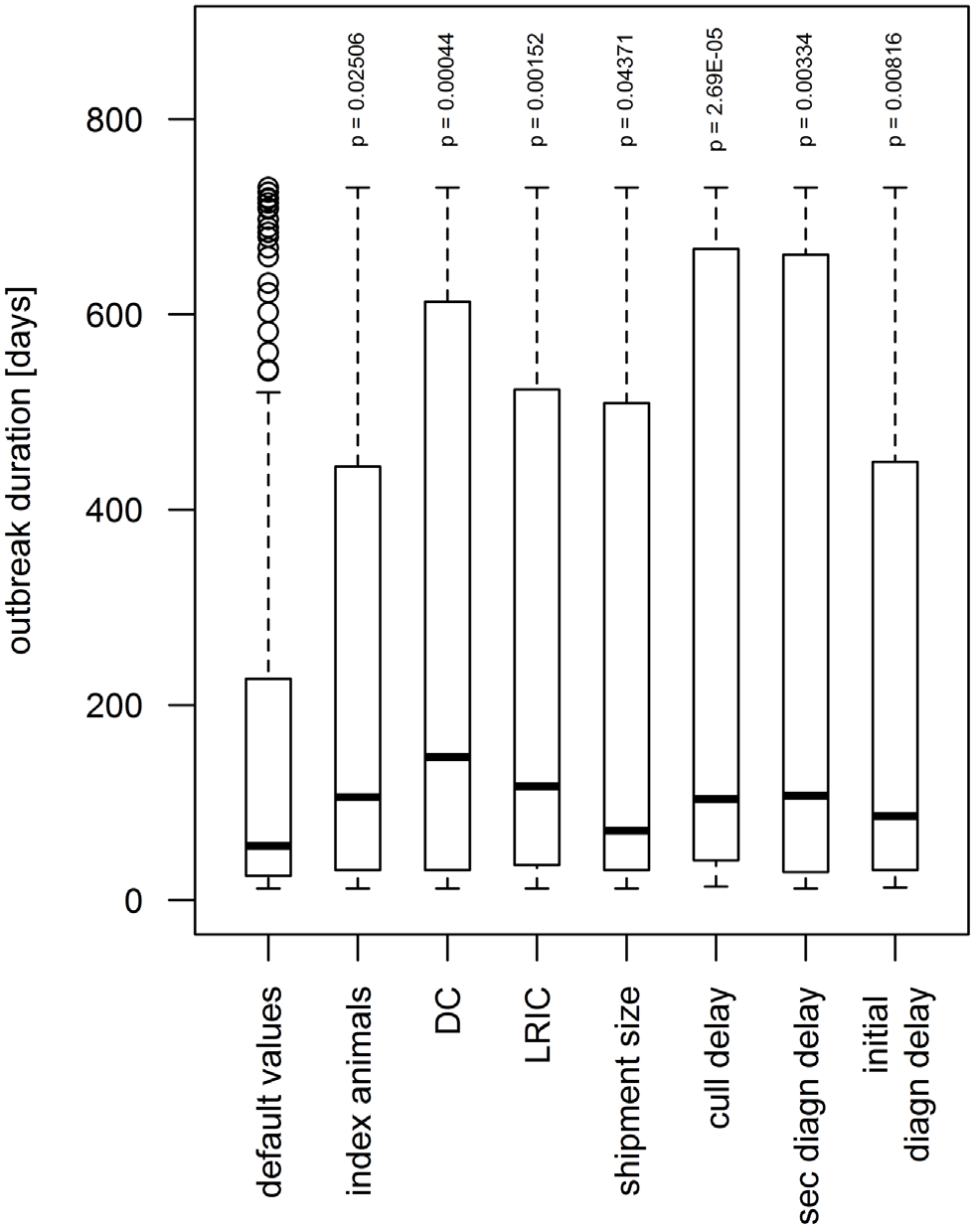
**Influence of model parameters found to be statistically significant (*p* < 0.05) on simulated outbreak duration during the sensitivity analysis**. When increasing the value by 15% above the default value, the outbreak duration augmented. The boxes represent the interquartile range with the median as black line, the whiskers extend to the most extreme data point that is no more than 1.5 times IQR from the box. Index animal, number of animals in the index premises; DC, direct contact frequency; LRIC, low-risk indirect contact frequency; shipment size, number of animals included in a DC; cull delay, delay between detection and depopulation of infected premises; initial/sec diagn delay, delay between start of clinical signs and detection of FMD on the premises for the index premises and secondary cases, respectively.

Seven parameters showed a significant influence on the simulated outbreak duration for the upper values, i.e., when values 15% higher than the default value were used (Figure 6). When the number of index animals was set to 120, which correspond to the number of pigs on the index farm, the median of the outbreak duration increased significantly from 56 to 106 days (*p* = 0.025). A higher shipment size showed a significant influence on the duration by increasing the median by 15 days (*p* = 0.044). When the diagnosis delay (i.e., the period between the start of clinical symptoms and the detection of FMD on the IP) of the index IP, the diagnosis delay of secondary IPs and the frequency of LRIC were set to 15% above their default value, the median outbreak duration increased to 86, 107, and 116, respectively (*p* < 0.01). A 15% higher frequency of DC and a longer delay between detection of FMD on the IP and culling of the animals (culling delay) increased the median to 146 days and 104 days, respectively (*p*-value < 0.0005).

**Figure 7:**
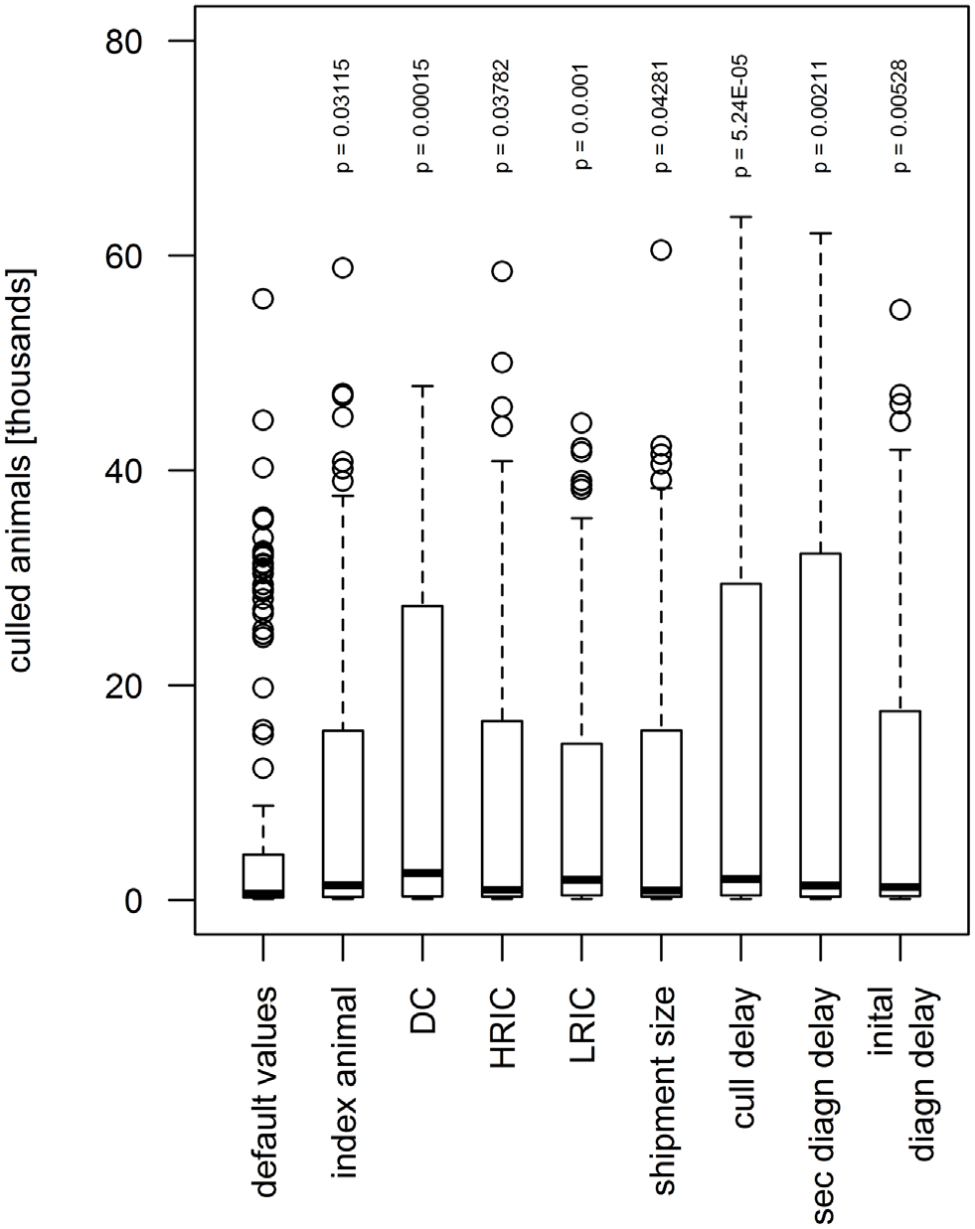
**Influence of model parameters found to be statistically significant (*p* < 0.05) on simulated number of animals culled during the sensitivity analysis**. When increasing the value by 15% above the default value, the number of animals culled augmented. The boxes represent the interquartile range with the median as black line, the whiskers extend to the most extreme data point that is no more than 1.5 times IQR from the box. Index animal, number of animals in the index premises; DC, direct contact frequency; HRIC, high-risk indirect contacts frequency; LRIC, low-risk indirect contact frequency; shipment size, number of animals included in a DC; cull delay, delay between detection and depopulation of infected premises; initial/sec diagn delay, delay between start of clinical signs and detection of FMD on the premises for the index premises and cases, respectively.

The increase of the values of eight parameters by 15% showed significant influence on the number of culled animals and premises (Figure 7). The shipment size, the number of index animals, and the frequency of HRIC significantly increased the median number of culled animals from 905 to 897, 1376, and 958, respectively, and the median of culled premises (20 in the default scenario) to 30, 42, and 28 (*p* < 0.05). When the diagnosis delay of the index IP, diagnosis delay of secondary IPs and the frequency of the LRIC increased by 15%, the number of culled animals augmented significantly to 1202, 1338, and 1872, respectively, and the number of culled premises to 40, 46, and 62 (*p* < 0.005). As for the outbreak duration, the culling delay and the frequency of DC showed the highest significant influence on the number of culled animals and premises (*p* < 0.0002). A longer culling delay and higher frequency of DC resulted in an increase of the median of culled animals to 1045 and 1603, respectively, and culled premises to 52 and 66.

For the 15% decrease of the default value and for all other parameters tested in the sensitivity analysis, no significant influence on the outbreak duration and number of culled animals and premises was detected (*p* > 0.05).

## Discussion

This study used historical data to reproduce a FMD outbreak with a modern epidemiological model to investigate the accuracy of disease spread predictions by this model. The research objective was not to achieve an improved fit to the outbreak data by optimizing the parameterization of the model. Instead, we used the best available data from historical and current literature, expert opinion, and contemporary witnesses as inputs – the same sources that would be available to inform parameter values for future epidemics. Therefore, we aimed to investigate how precise the model simulations are when parameterized according to best available knowledge, by comparing the simulations with the recorded data of the real outbreak.

The comparison of the simulated outbreaks and the real epidemic was mainly based on the number of culled animals, because data on animal level were more certain than those on premises level. This was because information on the number of animals of each species per community was known, whereas information on premises level was lacking for the year 1966 and the premises structure had to be reconstructed. In fact, the ratio of culled animals to depopulated premises in the model outcomes was found to be smaller than what was recorded during the real epidemic. Beside the issue of challenging premises reconstruction that might not fully represent the reality in 1966, this discrepancy of culled animal-IP ratio might also be caused by real reporting bias during the outbreak. Farmers with a larger premises size might have more likely recognized and reported the disease, or were more motivated to receive compensation for animal losses that requires reporting, which may result in underreporting for small IPs.

It was not possible to correctly reproduce the outbreak duration by the model. All simulations that approximated the real outbreak size by far exceeded the reported duration of the real epidemic. This was caused by cases occurring after the peak from week 21 onward when the real outbreak was declared to be controlled. Although this can be seen as a weakness of the model structure, the period of highest interest in model outputs is the earlier phase of the epidemic, in which critical control strategy decision are made (for example, whether to start vaccination).

The spatial spread of the 10% largest outbreaks well reflected the area infected during the real outbreak, although in 6 of those 15 largest simulated outbreaks the disease spread to parts in the east of Switzerland, which had not been infected in 1965/66 (Figure 3). Yet, the spatial distribution of the cases within the infected area differed from the real epidemic mostly by predicting three clusters of cases, where hardly any case was reported in 1965/66. These clusters are located in the southern, middle-western region of Switzerland, thus, in the alpine mountain area. Two explanations for this discrepancy are discussed. First, because the model did not consider the topography of Switzerland, and therefore, movements also occur across the Alps similarly as in the lowland, these areas had a higher chance to be infected in the simulated epidemics than in reality. Once FMD was spread to these regions, the number of cases heavily depended on the local density of premises, which was high in all of the three cluster regions (Figure S3 in Supplementary Material). Second, there may well be a recording bias during the 1965/66 outbreak with significant underreporting of cases in the Alps, because access to the mountain valleys was limited, particularly during winter time when the epidemic peaked.

The Swiss FMD outbreak in 1965/66 was larger in terms of animals slaughtered than most model simulations predicted, but in congruence with the 10% largest simulated outbreaks (Table 2). Reasons for the large epidemic observed compared to simulated model outbreaks can be categorized into the following sources: (a) the model structure and/or parameter value estimates were not accurate enough to reflect the reality, (b) reporting bias during the real outbreak distorted the number of slaughtered animals recorded, and (c) it may be the truth that the 1965/66 FMD epidemic in Switzerland was very large and it, therefore, should approximate the largest simulated outbreaks only.

Several issues in the model structure can be identified that suspect to cause smaller simulated outbreaks than observed in reality. First, the policy of slaughtering animals on IPs, which was applied in 1965/66, instead of culling and destroying on place, could have propagated the spread of FMD. The transportation of infectious animals to slaughterhouses allowed the virus to spread along traffic routes. In 1965/66, events were reported in which trucks, transporting infectious animals to the slaughter houses, had been the cause for the outbreak of FMD in premises near traffic routes (4). Another accumulation of outbreaks has been observed downstream slaughterhouses. Also, although the meat from infectious animals should have been treated with lactic acid before sold for consumption, it still remained a risk for further spread. Both of these possible pathways for FMD spread were not implemented in the model, where all animals on IP were considered to be culled on place.

Second, the model only allowed implementation of vaccination for specified premises types, but not for individual species within premises. Therefore, during the cattle vaccination campaign, every premises type with cattle was chosen for vaccination, knowing that pigs and small ruminants in mixed premises with cattle were simulated to be vaccinated as well. This caused simulated vaccination coverage in the livestock population higher than in reality. With the pig vaccination campaign that was simulated to start after the completion of cattle vaccination, this effect was even pronounced for the small ruminant species. This overestimation of the livestock vaccination coverage may have led to smaller modeled outbreaks than reality.

Third, the statutory provisions for movement restrictions (size and retention of surveillance and protection zones) were used in the model knowing that they were not always respected properly. The proportion of disrespect of movement restrictions was not known. Witnesses believed that farmer compliance was about 90% for DC, which was used as compliance level in the simulation model. For HRIC and LRIC, a lower value of 70–80% was used. As the real outbreak was bigger than the model predicted, it may be that farmer compliance was lower than 90%. This is supported by documents on criminal prosecutions in the States Archives of the canton of Zürich reporting that farmers mainly disrespected movement restrictions for family members. However, movement restrictions for animals seemed to have been well respected.

The second objective of the study was to investigate the effect of vaccination applied during the 1965/66 epidemic. Experience during the 1965/66 outbreak indicated that vaccination played a crucial role in stopping the epidemic (4). According to the model outputs vaccination does not provide much benefit for relatively small epidemics. However, when an outbreak becomes large, it was found that vaccination dramatically reduced the number of culled animals (Figure 4). These findings are consistent with the results of modeling the spread and control of FMD in the contemporary Swiss animal population, where vaccination was found to be effective to control large outbreaks (21). Evidence could, therefore, be gained for supporting the hypothesis that the mass livestock vaccination applied was pivotal to control the outbreak in 1965/66, as, according to the model outcomes, this epidemic was a large one. However, the definition of a threshold, probably a population-dependent one, above which an outbreak can be identified as large would be part of further investigation.

According to the model outputs, the outbreak duration was detected to be significantly longer when vaccination was not applied. However, this finding has to be interpreted with caution because of two reasons. First, four (2.6%) and 12 (8%) of the 150 simulations for the vaccination and non-vaccination strategy, respectively, reached the defined time limit of 730 days and they would have probably lasted longer. The dataset for the statistical analysis was, therefore, biased toward shorter outbreak durations, particularly for the non-vaccination strategy. Second, the model did not well reproduce the outbreak duration of the real epidemic, thus, for the vaccination strategy, and therefore the outbreak duration may be a non-ideal measurement to compare between strategies. Nevertheless, the model outputs still provided evidence that the number of long-lasting outbreaks is considerably higher for the non-vaccination than vaccination strategy (Figure 5).

As in all models, the reality can never be fully reflected. The epidemic curve of the 1965/66 outbreak showed three peaks (week 10, 12, and 15) (Figure 2). After detection and implementation of control measures resulting in a decline of the curve, the second peak was caused by feeding of contaminated skim milk to pigs and calves and the third peak by the “pig-FMD outbreak,” because pigs had not been vaccinated until this point in time (4). Because the simulation model applied here is a generic FMD model in which such specific situations were not implemented, these peaks could not be reproduced by the model. Also, the model samples contact premises randomly under consideration of contact probabilities between premises types and distance between premises. However, social and commercial networks for both, DC and H/LRIC that certainly exist, were not respected in the model. It is expected that incorporation of such networks would reduce the variation between simulations that would lead to more precise predictions. However, intensive data collection would be required to inform such country-wide networks.

Another factor not adequately represented in the model was the topography of Switzerland. In the implementation of movement bans and ring vaccination in 1965/66, the topography was a determining factor, as according to statutory provisions these control measures were organized “depending on the local conditions” (e.g., considering lakes, mountains, or rivers). In the model, these zones were simulated as rings and they may have, therefore, been smaller or larger than in reality.

The role of the small ruminants was a challenging to handle in the model as historical data were completely lacking for the 1965/66 outbreak. They had neither been vaccinated nor related cases reported. Possible reasons can be that (a) at that time, small ruminants were considered as not important for the propagation of the disease, (b) there were no resources left to report or cull them, or (c) they were not affected in this epidemic. Although little was known about the role of small ruminants in 1965/66, they have been included in the simulation model because they have been reported to play an important role in the propagation of FMD elsewhere (16, 29, 30). As possibility (a) and (b) were considered to be more likely and records of slaughtered small ruminants were lacking, the diagnosis delay for pure small ruminants premises was set at a value that they would had never been detected, but still may had spread the disease during the infectious period. Further investigation of the potential impact of small ruminants on this outbreak by using varying probability of detection for these premises could contribute to better define their role in the 1965/66 but also future epidemics.

The sensitivity analysis using a variability of 30% around the default value revealed seven parameters to influence the outbreak duration and an additional parameter to influence the outbreak size: the culling delay, the diagnosis delay for both first and secondary cases, the frequency of DC and LRIC, the shipment size, the number of index animals, and, only in terms of the outbreaks size, the frequency of HRIC. The significance of all these parameters can be well explained. The great influence of the culling delay is in accordance with experience from the Swiss outbreaks in the twentieth century. In all of the three epidemics that occurred in Switzerland in the twentieth century (1920/21, 1939/40, and 1965/66), slaughtering has been delayed because of limited resources that led to increased disease propagation. Resource allocation for and planning of rapid culling and disposal strategies may, therefore, be crucial during the preparedness phase. The significance of the detection delay of FMD epidemics and the diagnosis delay for secondary IPs is in accordance to other studies. For example, Carpenter et al. estimated the number of culled animals to increase by factors 7 and 25 when the diagnosis of the first case increases from 1 to 2 and 3 weeks, respectively (31). Keeping the awareness of FMD but also other exotic diseases high in the veterinary and agriculture sector may thus have a pivotal role on the size of future epidemics. A high rate of DC, indirect contacts, and a larger shipment size leads to a higher probability of dissemination of the virus. A higher number of index animals resulting in a higher excretion of virus leads to a higher probability to transport a diseased animal before the onset of clinical signs and, therefore, increases the propagation of FMD via DC. The results of the sensitivity analysis also help to identify crucial parameters for which values should be estimated as accurate as possible to improve preparedness. Particularly, the quality of preparedness would benefit from most accurate data on the movement frequencies from and to premises (i.e., frequency of DC, HRIC, and LRIC). The extend of how much the model would perform better in case of more accurate movement data is hard to estimate. While animal movements are recorded on animal level for cattle and on premises level for pig in Switzerland, comprehensive data on DC for small ruminants are currently lacking. It would be of high relevance, not only for the spread of FMD but also other infectious diseases, to establish a comparable database for sheep and goats.

Infectious disease models are often used to forecast the development of an ongoing epidemic and as a tool to support decision making. This study demonstrated that it is also useful as a tool to analyze previous or even historical outbreaks. However, it also confirmed that predicted epidemics from a model, even if it is parameterized most carefully, need not be correct in all aspects of epidemic sizes and duration. Often, the availability and quality of data required to reliably parameterize a simulation model are limited. Here, while data on FMD cases were relatively well recorded, data on the susceptible population was only partly available and premises included in the model had to be reconstructed. Nevertheless, it could be demonstrated that in the Swiss FMD outbreak in 1965/66, vaccination seems to have been the crucial step for disease control. With vaccination as an additional measure to conventional strategies like depopulation of IPs, movement bans, and disinfection, the epidemic was stopped in less than half a year, reducing the number of slaughtered animals significantly compared to a NV scenario. As such, further evidence could be provided to support the emergency vaccination strategy for expected large FMD outbreaks. It is, therefore, essential to keep FMD expertise and disease awareness alive, review personal and material resources to be prepared for future outbreaks, and reduce its impacts as much as possible.

## Author Contributions

DZ, GS-R, SH, and SD contributed to the study conception. DZ and SD modified the model code and DZ run the model. DZ, SH, and HS were involved in collecting historical data and analyze them. DZ wrote the first draft of the manuscript and all authors contributed to the final version and approved it for publication.

## Conflict of Interest Statement

The authors declare that the research was conducted in the absence of any commercial or financial relationships that could be construed as a potential conflict of interest.
