# Supplementary material for: Evidence for Emergency Vaccination Having Played a Crucial Role to Control the 1965/66 Foot-and-Mouth Disease Outbreak in Switzerland
Source: Front Vet Sci. 2015 Dec 14;2:72. doi: 10.3389/fvets.2015.00072 (PMC4677095; doi:10.3389/fvets.2015.00072)
Supplement: Supplementary file 1 [file Table_1.PDF]

Table S1: Model parameters and their values and sources of a FMD outbreak simulation model applied for an outbreak in Switzerland in 1965/66

Parameter	Description [dimension]	Value	Lower and upper value used in the sensitivity analysis	Source
**General parameters**				
simulation time	running time of one iteration [day]	730		to allow outbreaks larger than the real outbreak (160 days (1))
number of index herds	the total number of selected index herds [herd]	1 herd in Brent, canton of Vaud		(1);<br>Official outbreak report (Swiss Federal Statistical Office)
index animals per herd	number of index animals per index herd [animal]	100	Lower: 80<br>Upper: 120 (all pigs)	assumption
initial diagnosis delay	time delay between appearance of first clinical animal in a herd and the diagnosis of infection for the first infected herd of an outbreak [day]	7	Lower: 6<br>Upper: 8	expert opinion, minutes of the OIE[(1)] conference in Rome (March 1966)
second diagnosis delay	time delay between appearance of first clinical animal in a herd and the diagnosis of infection for the secondly infected herds [day]	cattle and pig: pert-distribution (2,3,5)<br><br>small ruminants: pert-distribution (750,770,790)	cattle and pig:<br>Lower: pert-distribution (1,2,4)<br>Upper: pert-distribution (3,4,6)<br><br>small ruminants:<br>Lower: pert-distribution (637, 654,671)<br>Upper: pert-distribution (862, 885, 908)	Cattle and pig: expert opinion;<br>Small ruminants: we assumed that they will never be detected based in the fact that they were not mentioned in historical records

Parameters of the intra-herd spread

K	intra herd interaction rate: number of effective transmission contacts per infectious individual and day within the herd [animal/day]	dairy and beef herds and mixed herds without pigs: 4.125<br><br>pig herds and mixed herds with pigs: 25.79<br><br>small ruminant herds: 10.01		(2); for mixed herd with pigs the pig value was taken assuming worse case; for mixed herds without pigs the cattle value was taken due to the majority of cattle in this herd type (only 2% of this herd type are cattle free)
N	median of the herd sizes of the given herd type [animal]	for all herd types: 125		(2)
duration of latent period (infected but not infectious)	vector with each value representing the day (starting at day 1) after the animal has reached the latent state with the probability that the animal leaves the latent state and moves to the subclinical state on the next day [dmnl][(2)]	Cattle and mixed herds with cattle: (0.2,0.2,0.2,0.2,0.2)<br><br>pig and mixed herds with pigs:<br>(0.33,0.33,0.34)<br><br>small ruminant herds:<br>(0,0,0.166,0.166,0.166,<br>0.166,0.166,0.17)		(3,4)
duration of subclinical period	vector with each value representing the day (starting at day 1) after the animal has reached the subclinical state with the probability that the animal leaves the subclinical state and moves to the clinical state on the next day [dmnl]	(0,1),<br>i.e. the animals leave the subclinical state on day 2		(3,4)

duration of clinical period	vector with each value representing the day (starting at day 1) after the animal has reached the clinical state with the probability that the animal leaves the clinical state and moves to the immune state on the next day [dmnl]	All but pig herds:<br>(repeat 0 for 30 days, 1),<br>i.e. the animals leave the clinical state on day 31<br><br>Pig herds:<br>(repeat 0 for 20 days, 1),<br>i.e. the animals leave the clinical state on day 21		(3,5)
duration of immune period	vector with each value representing the day (starting at day 1) after the animal has reached the immune state with the probability that the animal leaves the immune state and moves to the latent state on the next day [dmnl]	for all herd types:<br>(repeat 0 for 750, 1),<br>i.e. the animals leave the immune state on day 751		by using this values no animal will leave the immune state for the entire simulation time

<u>Parameters of the four types of inter-herd spread</u>

daily DC	number of daily direct contacts from and to a herd [herds]	cattle herds and mixed herds with cattle: 0.14<br><br>pig herds, small ruminant herds and mixed herds without cattle: 0.01	Lower: 0.119<br>Upper: 0.161<br><br>Lower: 0. 0085<br>Upper: 0.0115	expert opinion
shipment size	number of individuals per shipment in a DC	pig and sheep herds: 20<br>mixed sheep-pig herds: 10<br>cattle, goat, mixed sheep-goat herds and mixed herds with two species, of which one is cattle: 5<br>mixed pig-goat herd: 7	Lower: 17, upper: 23<br>Lower: 8, upper: 12<br><br>Lower: 4, upper: 6<br>Lower: 6, upper: 8	expert opinion

		pig-sheep-goat herds: 4<br>other 3-species herds: 2<br>herds with all 4 species: 1	Lower: 3, upper: 5<br>Lower: 1, upper: 3<br>Lower: 0, upper: 2	
distance distribution DC	distribution function of the distances for direct contacts [km]	Herds with cattle: Weibull-distribution (shape=1, scale=72.5)	Lower: Weibull-distribution (shape=1, scale=61.5)<br>Upper: Weibull-distribution (shape=1, scale=83.3)	expert opinion, outbreak data
		Herds without cattle: Weibull-distribution (shape=1.2, scale=60)	Lower:<br>Weibull-distribution (shape=1.2, scale=54.3)<br>Upper:<br>Weibull-distribution (shape =1.2, scale=73.4)	
between herd shipment proportions	relative number of direct contacts received by herds of type *B* among all direct contacts sent out by herds of type *A* [dmnl]	direct contact are possible with the same probability (p=1) between herd types containing the same species, i.e. between the equal herd types, cattle – mixed herds with cattle, small ruminants – mixed herds small ruminants and pig – mixed herds with pigs<br><br>direct contact are impossible between others (p=0)		assumption
network probability	probability that a direct contact will be recorded and the contact herd identified as infected (traceability) [dmnl]	0.9		expert opinion
daily HRIC	daily number of high risk indirect	all herd types: 0.148	Lower: 0.126	expert opinion

	contacts from an infected herd [herd]		Upper: 0.17	
effectivity of HRIC	probability that a high risk indirect contact with an infected herd leads to disease transmission	pert-distribution (0.11,0.26,0.41)		(3)
distance distribution HRIC	distribution function of the distances for high risk indirect contacts (HRIC) [km]	integrated as step-function with half of the distances are being sampled from 0-4km, ¼ between 4-25km and 26-50km, respectively<br><br>Uniform-distribution (min =c(0,2,4,25) , max = c(2,4,25,50))	Lower:<br>Uniform-distribution (min =c(0,1.7,3.4,21.25) , max = c(1.7,3.4,21.25,42.5))<br><br>Upper:<br>Uniform-distribution (min =c(0,2.3,4.6,28.75) , max = c(2.3,4.6,28.75,57.5))	expert opinion
between herd HRIC proportions	relative number of high risk indirect contacts received by herds of type *B* among all direct contacts sent out by herds of type *A* [dmnl]	from cattle herds to mixed cattle herd=1, to other herds = 0.9<br><br>from small ruminant (sr) herds to:<br>mixed sr herds =1, to other herds =0.9<br><br>from pig herds to mixed pig herds = 1, to other herds = 0.9		expert opinion and outbreak reports
daily LRIC	daily number of low risk indirect contacts from an infected herd [herd]	herds with cattle: 4<br>herds without cattle: 2	Lower: 3.4, upper: 4.6<br>Lower: 1.7, upper: 2.3	expert opinion
effectivity of	probability that a low risk indirect	pert distribution		(3)

LRIC	contact with an infected herd leads to disease transmission	(0.005,0.0525,0.1)		
distance distribution LRIC	distribution function of the distances of low risk indirect contacts (LRIC) [km]	Uniform-distribution (min=0, max=10)	Lower : Uniform-distribution (min =0, max=8.5) Upper : Uniform-distribution (min=0, max=11.5)	(3)
between herd LRIC proportions	relative number of indirect contacts received by herds of type *B* among all contacts sent out by herds of type *A* [dmnl]	cattle herds to cattle herds: 1 cattle herds to sr herds: 0.8 cattle/ sr /pig herds to pig herds: 0.5		expert opinion
distance distribution LAS	distribution function of the local area spread (LAS) depending on the maximal distance from the infectious herd to the contact herd where infection can be successful [km] and the daily probability that any herd in this circle will be infected by the infectious herd [dmnl]	maximal distance: 2 km, daily probability: 0.0012		(3)
**<u>Parameters of the control strategies (movement restrictions and vaccination)</u>**				
size of movement restriction zones	radius of surveillance zone (SZ) and infected area (IA) [km]	SZ: 6 IA: 4		expert opinion, outbreak reports
durations of surveillance zone (SZ) and infected	time from slaughter of infected herd that triggered a SZ or IA to lifting of the zone; movement restrictions gets active one day	SZ: 21 IA: 21		expert opinion, outbreak reports, minutes of conferences of cantonal veterinarians

area (IA)	after the diagnosis of the infected herd [day]			
control function	function of the farmers' compliance for movement restrictions for direct (DC) and indirect (IC) contacts as distribution of proportional reduction of these contacts in the surveillance zone and infected area [dmnl]	DC: pert-distribution (0.9,0.95,1)<br><br>IC in SZ: pert-distribution (0.5,0.7,95)<br><br>IC in IA: pert-distribution (0.5,0.8,0.98)		expert opinion
vaccination delay	time between detection of the infected herd which cause the vaccination and protective effect of vaccine, including time between detection of the disease until vaccination is applied (application delay) and time between vaccination and protection of the herd (protection delay) [day]	application delay = 8<br>protection delay = 14	Lower: 19<br>Upper: 25	application delay: assumption<br>protection delay: (6–8)
vaccine production time	time between detection of the first case and when vaccines are delivered for immediate application [day]	14		assumption
vaccine efficacy	vaccine efficacy on herd level after the completion of the vaccination delay [dmnl]	Uniform-distribution (0.8,0.9)	Lower: Uniform-distribution (0.68, 0.75)<br>Upper: Uniform-distribution (0.92, 1)	expert opinion, minutes of conference of cantonal veterinarians
vaccination	proportional reduction of	0.35		(9)

reduce	infectivity of an animal that has been vaccinated less vaccination delay days prior to infection [dmnl			
vaccination radius	radius of the circle around each infected and detected herd within each herd is submitted for vaccination [km]	5		expert opinion
larger vaccination radius	implementation of a larger vaccination radius from a certain point in time [km]	25		(1)
time to extended vaccination cattle/pig	the vaccination radius will be changed to “larger vaccination radius” at this point in time [day]	55 (cattle);<br>85 (pigs)		(1)
larger vaccination radius2	implement of a larger vaccination radius from a certain point in time [km]	400 (whole country)		(1)
time vacc all cattle (or pig)	the vaccination radius will be changed to “larger vaccination radius2” at this point in time [day]	84 (cattle);<br>100 (pig)		(1)
culling duration	distribution of time from diagnosis of the infected herd to the finalized depopulation in this herd [day]	Uniform-distribution (2,3)	Lower: Uniform-distribution (1,2)<br>Upper: Uniform-distribution (3,4)	expert opinion
threshold number	number of animals to cull per day which exceeded personal	200		expert opinion

animals to cull	and/or material resources for culling; results in a longer culling duration			
longer culling duration	culling duration when threshold number animals to cull exceeded	Uniform-distribution (5,6)	Lower: Uniform-distribution (4,5)<br>Upper: Uniform-distribution (6,7)	expert opinion
vaccTypes; vaccTypes Plus	herd type that was submitted for vaccination; all animals on the defined herd were vaccinated [dmnl]	herds with cattle (vaccTypes);<br>from day 85 onwards herds with cattle and pig (vaccTypesPlus)		(1)

(1) World Organization for Animal Health
(2) dimensionless

Reference list

1. Nabholz VA. [Foot-and-mouth disease 1965-1966 in Switzerland]. Schweiz Arch Tierheilkd. (1966) 108(12):717–28.

2. Bates TW, Thurmond MC, Carpenter TE. Description of an epidemic simulation model for use in evaluating strategies to control an outbreak of foot-and-mouth disease. AmJ Vet Res. (2003) 64(2):195–204.

3. Bachmann I. Assessing of the efficiency of different control strategies against Foot-and-mouth disease in Switzerland using a dynamic simulation model. University of Bern (2004)

4. Mardones F, Perez A, Sanchez J, Alkhamis M, Carpenter T. Parameterization of the duration of infection stages of serotype O foot-and-mouth disease virus: an analytical review and meta-analysis with application to simulation models. Vet Res (2010) 41(4):45.; doi: 10.1051/vetres/2010017

5. Burrows R. Excretion of Foot-And-Mouth Disease Virus Prior to Development of Lesions. Veterinary Record (1968) 82(13):387–8.

6. Orsel K, de Jong MCM, Bouma A, Stegeman JA, Dekker A. The effect of vaccination on foot and mouth disease virus transmission among

dairy cows. Vaccine (2007) 25(2):327–35.; doi: 10.1016/j.vaccine.2006.07.030

7. Orsel K, Dekker A, Bouma A, Stegeman JA, de Jong MCM. Quantification of foot and mouth disease virus excretion and transmission within groups of lambs with and without vaccination. Vaccine (2007) 25(14):2673–9.; doi: 10.1016/j.vaccine.2006.11.048

8. Aggarwal N, Zhang Z, Cox S, Statham R, Alexandersen S, Kitching RP, et al. Experimental studies with foot-and-mouth disease virus, strain O, responsible for the 2001 epidemic in the United Kingdom. Vaccine (2002) 20:2508–15.

9. Schley D, Paton DJ, Cox SJ, Parida S, Gubbins S. The effect of vaccination on undetected persistence of foot-and-mouth disease virus in cattle herds and sheep flocks. Epidemiol Infect. (2009) 137(10):1494.; doi: 10.1017/S0950268809002349
