# Supplementary material for: Evidence for Emergency Vaccination Having Played a Crucial Role to Control the 1965/66 Foot-and-Mouth Disease Outbreak in Switzerland
Source: Front Vet Sci. 2015 Dec 14;2:72. doi: 10.3389/fvets.2015.00072 (PMC4677095; doi:10.3389/fvets.2015.00072)
Supplement: Supplementary file 3 [file Image_2.PDF]

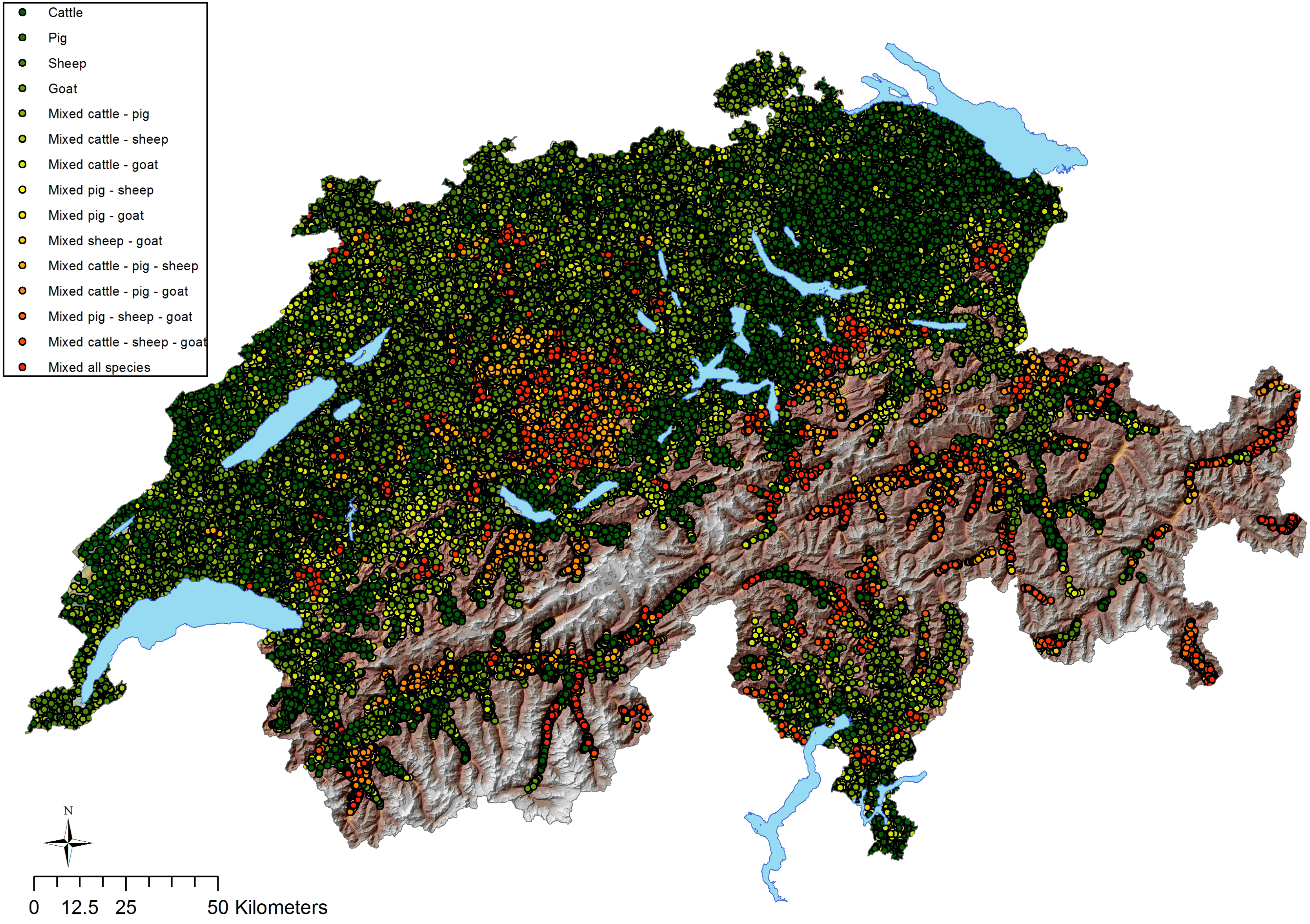

Figure S2: Map of Switzerland (including lakes) with the distribution of premises types of the reconstructed live-stock premises in 1965/66.
